# Enhanced thermoelectric performance of yttrium-doped ZnO ceramics *via* secondary phase formation and conventional sintering

**DOI:** 10.1039/d5ra03749b

**Published:** 2025-09-12

**Authors:** Aisha Saleem, Sajid Butt, Muhammad Irfan, Muhammad Faizan Masoud, Sumayya, Muhammad Umer Iqbal, Naseem Iqbal, Abdul Faheem Khan, Muhammad Abdul Basit

**Affiliations:** a Department of Materials Science and Engineering, Institute of Space Technology Islamabad 44000 Pakistan; b Department of Space Science, Institute of Space Technology Islamabad 44000 Pakistan sajid.butt@ist.edu.pk; c US – Pakistan Center for Advanced Studies in Energy, National University of Sciences and Technology Islamabad Pakistan

## Abstract

Zinc oxide (ZnO)-based ceramics have been widely studied for thermoelectric applications due to their abundance, non-toxicity, cost-effectiveness, thermal stability, and high Seebeck coefficient. In this work, a series of yttrium (Y)-doped ZnO samples was synthesized using the sol–gel method followed by conventional sintering. The thermoelectric property measurements coupled with detailed structural characterization were systematically performed to establish a structure–property relationship. The X-ray diffraction (XRD) analysis confirms limited substitution of Y at the Zn-site in the lattice of ZnO. The surplus Y-doping results in the formation of the secondary phase Y_2_O_3_. The sample with composition Zn_0.98_Y_0.02_O exhibited the highest power factor and figure of merit (*ZT*) values of 0.47 μW cm^−1^ K^−2^ and 5.6 × 10^−5^, respectively, at 575 K. The outlined study elucidates the effects of Y-doping in ZnO to understand the underlying transport phenomenon in ZnO ceramics.

## Abstract 2

Zinc oxide (ZnO)-based ceramics have been widely studied for thermoelectric applications due to their abundance, non-toxicity, cost-effectiveness, thermal stability, and high Seebeck coefficient.

## Graphical abstract

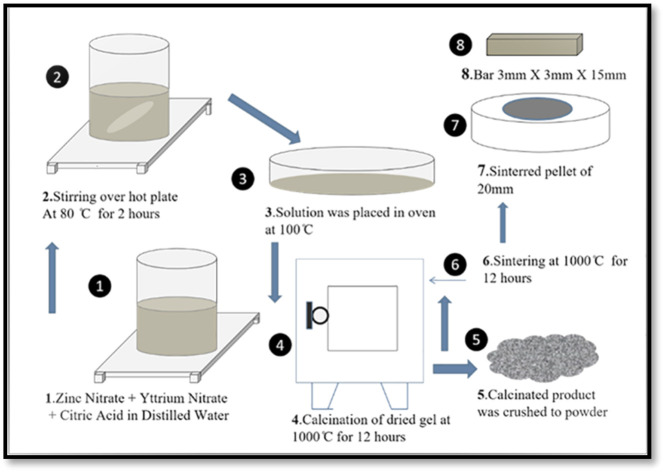

## 1 Introduction

Traditional energy sources such as fossil fuels have long been the primary means of meeting global energy demands; however, they pose a significant environmental risk due to the release of carbon dioxide. Reports indicate that nearly 60% of the energy produced in systems like those in the U.S. is lost as waste heat.^1^ These environmental consequences necessitate the exploration and adoption of alternative energy sources that offer cleaner and more sustainable methods for meeting our energy needs. Thermoelectric (TE) materials, which enable direct conversion of thermal energy into electricity and *vice versa*, offer a promising pathway to recover waste heat.^2,3^ TE devices offer a promising avenue for reducing our reliance on conventional energy and minimizing the negative environmental consequences. TE materials can convert waste heat directly into electricity.^4,5^ TE devices work without any mechanical parts like other conventional systems, and offer a more promising technique due to quietness and compactness.^6,7^ The conversion efficiency of any TE material is evaluated using a dimensionless figure-of-merit: 
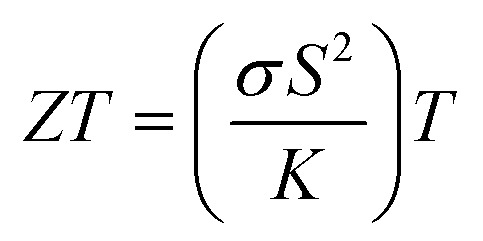
, where *σ*, *S*, *k* and *T* are the electrical conductivity, Seebeck coefficient, total thermal conductivity, and operating temperature. Furthermore *k* is a sum of electronic thermal conductivity (*k*_e_) and lattice thermal conductivity (*k*_l_). Due to the interplaying nature of *σ* and *S*, it is challenging to attain a high *ZT* value merely by increasing carrier concentration.^8,9^ Furthermore, (*σS*^2^) is explicitly defined as power factor (PF), so a high PF value is achieved through tuning carrier concentration, introduction of point defects through doping, and tuning of band structure ^10–13^ and compositing.^14^

Promising thermoelectric materials should exhibit (*ZT*) values greater than 1.^15^ Recent efforts have focused on developing environmentally friendly, low-cost, non-toxic, and thermally stable TE materials.^16–19^ Transition metal dichalcogenides, with their unique 2D layered structures, are among the leading candidates.^20–26^ Notable examples include PbTe-based alloys (*e.g.*, PbTe–MgTe–Se) with *ZT* ≈ 2.2 at 820 K^27^ and K-doped PbTe_0.7_S_0.3_ achieving *ZT* > 2 between 673–923 K.^28^ Cadmium, Cd-doped Ag_2_Se, shows *ZT* = 1.57 at 398 K, suitable for low-temperature applications.^29^ While high-entropy sulfides like Cu_7_Mg_2_Sn_2_ ZnSiS_13_ offer both thermal stability and *ZT* > 1.^30^ Currently, chalcogenides such as SnSe,^31^ Cu_2−*x*_Se,^23,32,33^ PbSe,^34^ and Bi_2_Te_3_ (ref. 35–37) are getting attention due to their high (*ZT*) values. Recently thin films have emerged as promising (TE) materials due to their enhanced power factors and *ZT* values, though challenges remain regarding their thermal and chemical stability.^24,32,38,39^ Therefore, oxide-based thermoelectric (TE) materials are considered a potential candidate within the TE research community due to their non-toxicity, cost-effectiveness, and ease of processing.^37,40–42^ Thus, various oxides such as ZnO,^43^ SrTiO_3_,^8^ Ca_3_Co_4_O_9_,^44^ BiCuSeO,^45^ and LaCoO_3_ (ref. 14) have been extensively studied for thermoelectric applications.

ZnO is an n-type thermoelectric material that has received special attention from the community due to its natural abundance, cost-effectiveness, non-toxicity, and thermal and chemical stability.^41,46,47^ ZnO is a direct band gap semiconductor, having a band gap value of 3.3 eV, and its band gap can be reduced through elemental doping, which helps to improve its electrical conductivity.^48^ Undoped ZnO, attributed to Zn interstitials, O vacancies, and inherently native defects, leads to higher electrical conductivity of ZnO.^49–51^ There have been various studies conducted on elemental doping into ZnO, including Bi,^52^ Al,^46,52–56^ In,^57–59^ Co,^60^ Ni,^61,62^ Mn,^63^ Fe,^43,64–67^ Sn,^68^ Sb^69^ and Sb, Sn doping^39,70,71^ have improved thermoelectric performance. In contrast, Al and Ga-doped ZnO prepared *via* sol–gel routes improved electrical conductivity but reduced Seebeck coefficients, leading to only marginal PF gains.^72^

In the present study, a facile sol–gel route has been employed to partially substitute yttrium (Y) at the Zn-site of ZnO to enhance its thermoelectric performance. Incorporation of trivalent dopants such as yttrium (Y^3+^) into the ZnO lattice is a well-established method to improve its electrical conductivity by increasing the electron carrier concentration.^73^ Due to the intrinsically low carrier density of pristine ZnO, its conductivity is limited. Substitution of Zn^2+^ by Y^3+^ donates excess electrons, thereby increasing carrier concentration and facilitating enhanced charge transport. Y-doped ZnO nanorods, used in quantum dot-sensitized solar cells (QDSC) photoanodes, promote nanorod growth and enhance quantum dot loading due to increased surface area. Y-doped in ZnO also reduces electrical resistance and surface defects, leading to increased electron mean free path.^74^ Comprehensive structural characterizations were conducted to elucidate the underlying conduction mechanisms within the resulting multiphase system comprising Y_2_O_3_ and Zn_(1−*x*)_Y_*x*_O.

## 2 Experimentation

### 2.1 Materials and methods

Pure and Y-doped ZnO samples having the stoichiometry of Zn_1−*x*_Y_*x*_O (where *x* = 0.01, 0.02, and 0.03, corresponding to Y-1%, Y-2%, and Y-3%, respectively) were synthesized using the sol–gel method. In this process, stoichiometric amounts of zinc nitrate hexahydrate (Zn(NO_3_)_2_·6H_2_O) and yttrium nitrate hexahydrate (Y(NO_3_)_3_·6H_2_O) were dissolved in distilled water. The chemical equation is given below as eqn (1). Citric acid was added as a chelating agent to ensure uniform distribution and complexation of metal ions. The solution was stirred at 80 °C for 2 hours for homogeneity.

After stirring, the solution was dried at 120 °C for 8 hours to obtain a spongy gel, which was ground into a fine powder. This powder was calcined at 1000 °C for 12 hours in air to induce crystallization and remove organic content. The eqn (1) given below describes the formation of Y-doped ZnO from zinc nitrate and yttrium nitrate hydrates by thermal decomposition, with gaseous by-products (NO_2_, H_2_O, O_2_).1
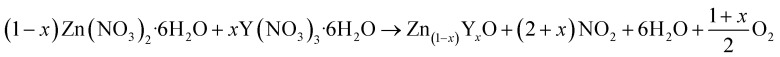


**Fig. 1:**
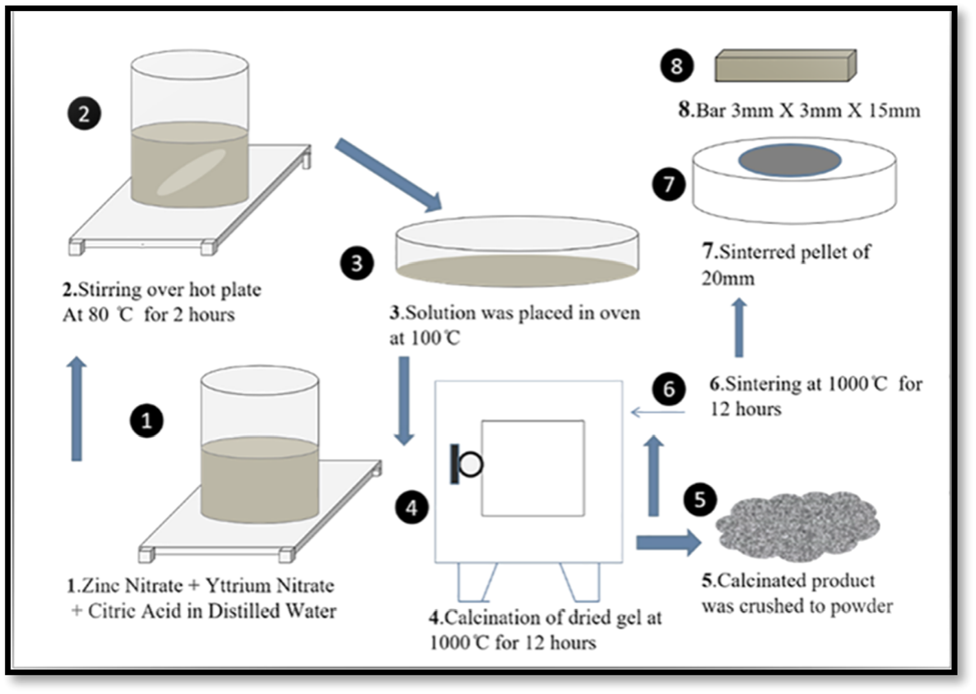
Schematic diagram demonstrating the synthesis route for pure and yttrium-doped ZnO ceramics through the sol–gel process.

The calcined powder was re-ground and pressed into pellets with a diameter of 20 mm under the uniaxial pressure of 70 MPa for 5 minutes. The obtained pellets were then sintered at 900 °C for 8 hours to enhance grain growth, crystallinity, and densification. Finally, the sintered pellets were cut into rectangular bars with dimensions of 3 × 3 × 15 mm^3^ for thermoelectric characterizations, as shown in Fig. 1.

The sol–gel technique is a cost-effective and scalable method for synthesizing doped ZnO, offering precise stoichiometric control, molecular-level dopant distribution, and excellent compositional uniformity. It requires minimal equipment and produces dense, smooth materials suitable for both laboratory and industrial applications.^75^

Room-temperature crystal structure analysis is carried out using X-ray diffraction (XRD) with Cu Kα radiation (*λ* = 0.15406 nm), employing a D8 Advance diffractometer (Bruker, Germany). The surface morphology and microstructural features are examined using Scanning Electron Microscopy (SEM, TESCAN Mira-3), which is also equipped with an energy-dispersive X-ray spectroscopy (EDS) detector for elemental composition analysis. The temperature-dependent electrical conductivity (*σ*) and Seebeck coefficient (*S*) are simultaneously measured, and the calculated power factor (PF) is calculated using a thermoelectric parameter measurement system (Joule Yacht-NAMICRO-3L) based on the four-probe method.

## 3 Results and discussion

### 3.1 Structural properties

**Fig. 2:**
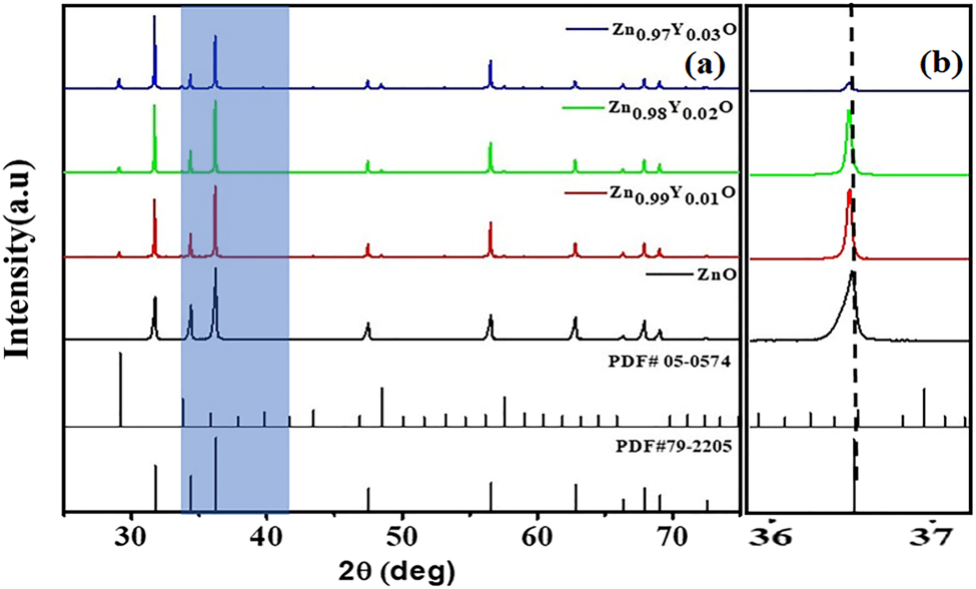
(a) XRD spectra of ZnO, Zn_0.99_Y_0.01_O, Zn_0.98_Y_0.02_O, and Zn_0.97_Y_0.03_O showing phase evolution and crystallinity. Standard JCPDS patterns for ZnO (01-079-2205) and Y_2_O_3_ (05-0574) are included for reference. (b) Magnified image of peaks shifting.

**Fig. 3:**
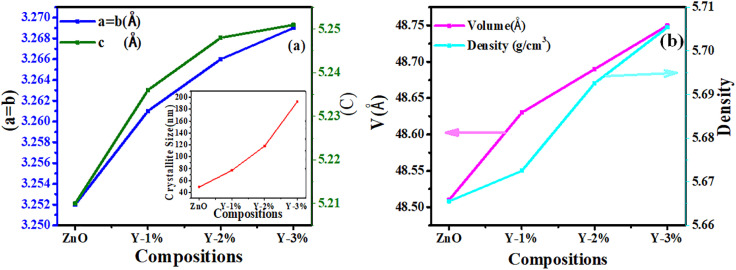
(a) Lattice parameters *a* = *b* and *c* of ZnO and Y-doped ZnO as a function of yttrium (Y) concentration, showing a gradual increase with doping, where, crystallite size *vs.* doping concentration is given in the inset. (b) Unit cell volume and theoretical density of ZnO and Y-doped ZnO, both showing a rising trend with increasing *Y* content.

The XRD patterns of pristine and Y-doped ZnO (Zn_(1−*x*)_Y_*x*_O), presented in Fig. 2, confirm the hexagonal wurtzite phase of ZnO (PDF #79-2205). A slight 2*θ* shift upon Y doping implies partial substitution of Y^3+^ into the ZnO lattice. However, the detection of Y_2_O_3_ reflections (PDF #05-0574) confirms the formation of a secondary phase, indicating that Y incorporation exceeds the solubility limit in the ZnO host lattice.^47,76,77^ The lattice parameters were measured using the least-squares refinement method, and it can be observed that both lattice parameters *a* and *c* increase with increasing concentration of Y content due to the larger ionic radius of Y than that of Zn, as shown in Fig. 3(a). Some previous studies ^78,79^ has been shown that the replacement of Zn by metal ions with a higher ionic radius increases the lattice size. Thus, due to a mismatch of the ionic radii of Zn^+2^ (0.74 Å) and Y^+3^ (0.89 Å) leads towards limited doping of Y into the lattice of ZnO, and surplus Y is crystallized as Y_2_O_3_. One report has also suggested the formation of a pure Y phase segregated into ZnO upon doping.^80^ Furthermore, after Y-doping, a systematic expansion in crystallite size has been observed, as shown in the inset of Fig. 3(a), which suggests that Y-doping has promoted grain growth. On the other hand, Y-doping results in a simultaneous increase in the unit cell volume and theoretical density, as shown in Fig. 3(b), which is likely to result in a suppressed phonon transportation in the samples.

**Fig. 4:**
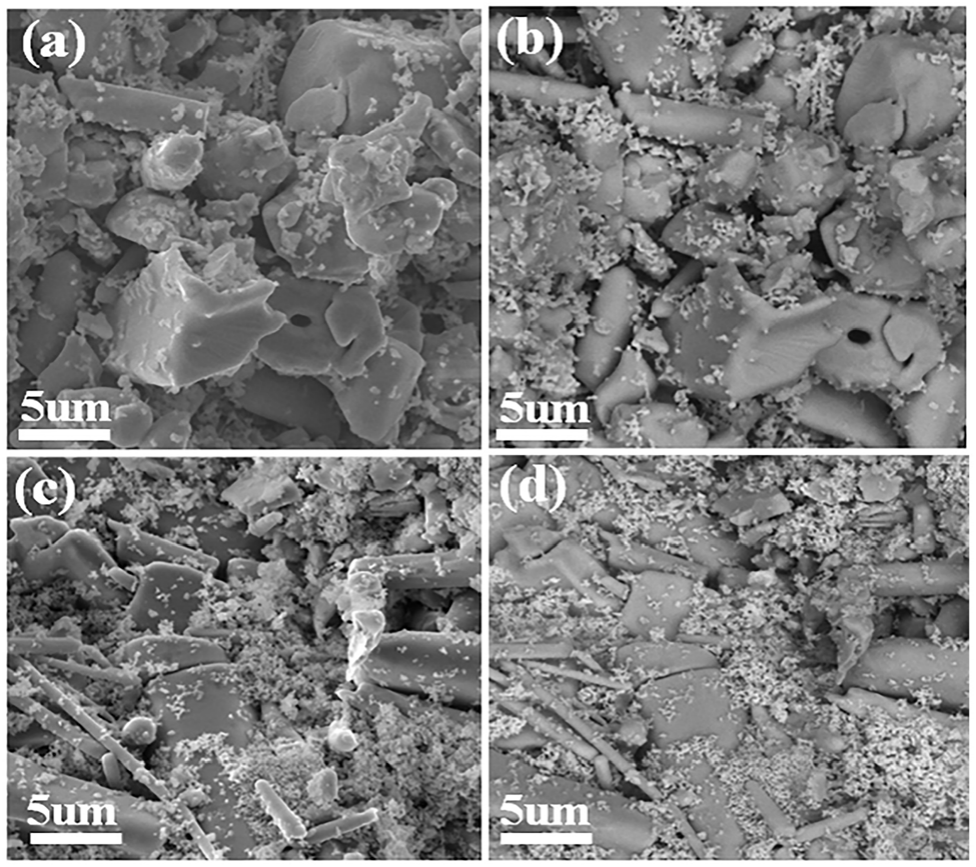
Secondary electron (a) and BSE (b) images of the Zn_0.99_Y_0.01_O (Y-1%), and the corresponding images for the Zn_0.97_Y_0.03_O (Y-3%) sample are given in (c) and (d), respectively.

The SEM images representing surface topography and phase contrasts were recorded using secondary electrons (SE) and backscattered electrons (BSE), respectively, as shown in Fig. 4. The (SE) and (BSE) images of the (Y-1%) sample are shown in Fig. 4(a) and (b), respectively, while the corresponding images for the (Y-3%) sample are presented in Fig. 4(c) and (d), respectively. The bigger rodlike grains belong to the Y-doped ZnO phase. BSE images recorded for (Y-1%) and (Y-3%) reveal bright and dark contrasts, revealing the presence of two phases. The smaller nanoparticles dispersed over the grains and grain boundaries are identified as Y_2_O_3_, as indicated by the bright contrast in the BSE images shown in Fig. 4(b) and (d). This contrast arises from the higher average atomic weight of Y_2_O_3_ compared to Y-doped ZnO. The presence of the Y_2_O_3_ phase, as confirmed by XRD analysis, suggests a possible saturation limit of Y in the lattice of ZnO.

**Fig. 5:**
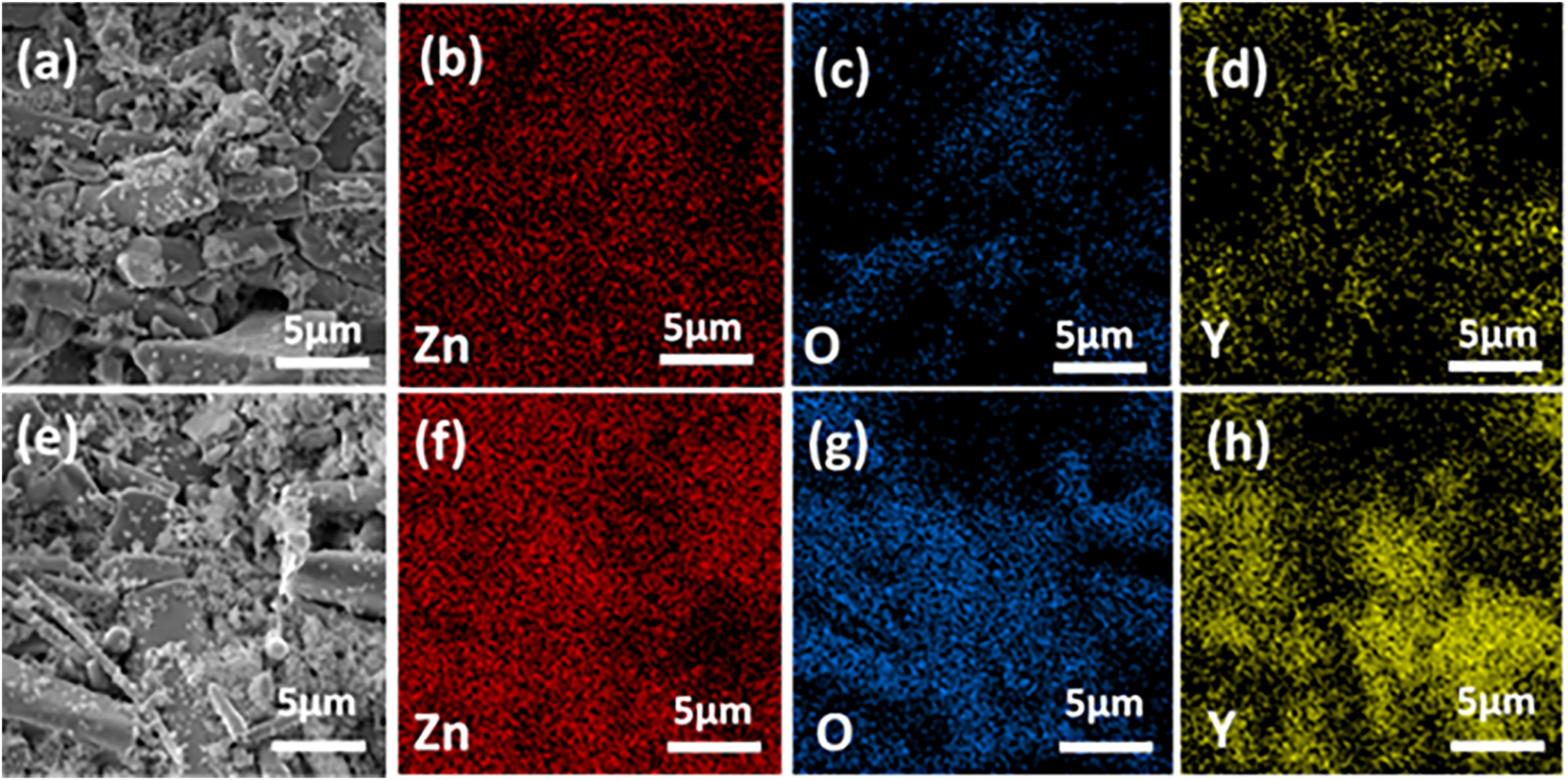
Backscattered electron (BSE) image (a) along with EDS elemental mapping (b–d) of Zn_0.98_Y_0.02_O, and BSE image (e) along with EDS elemental mapping (f–h) of Zn_0.97_Y_0.03_O.

**Fig. 6:**
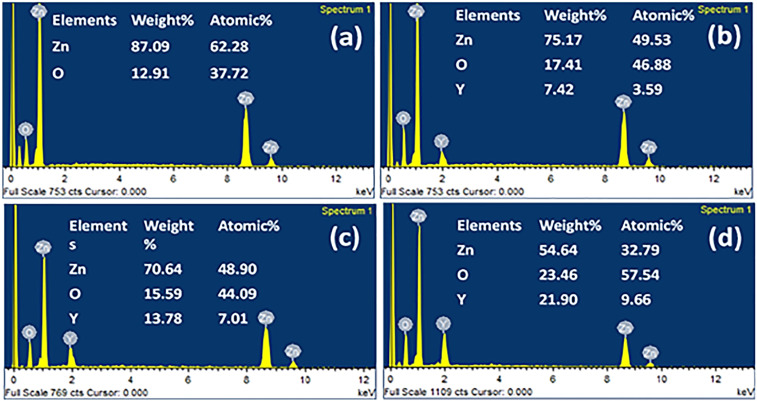
Energy-dispersive spectroscopy data showing atomic and weight percentages of (a) ZnO and yttrium-doped zinc oxide (b) Zn_0.99_Y_0.01_O, (c) Zn_0.98_Y_0.02_O, (d) Zn_0.97_Y_0.03_O, respectively.

Multiphase compositions were further confirmed by EDS mapping for the typical compositions of (Y-2%) and (Y-3%), as shown in Fig. 5(a)–(h). While looking at the elemental distributions, it can be concluded that nanoparticles, as represented by bright contrasts in BSE images as shown in Fig. 5(a) and (e), are Y_2_O_3_. The elemental composition of all the series of pure and Y-doped samples is shown in Fig. 6(a)–(d). All the doped samples have demonstrated a gradually increased concentration of *Y* upon increasing doping concentration.

### 3.2 Thermoelectric transport parameters

**Fig. 7:**
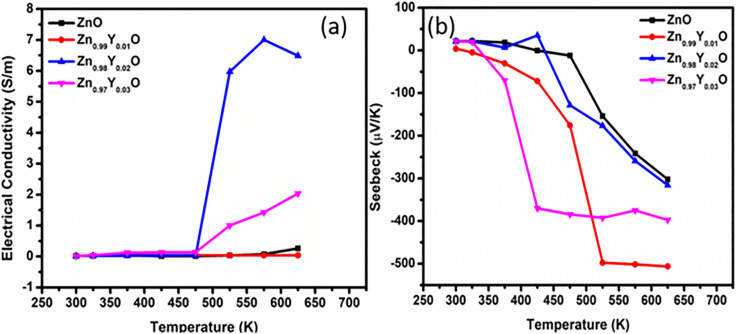
Electrical conductivity (a) and Seebeck coefficient (b) of ZnO and Y-doped ZnO (Zn_0.99_Y_0.01_O, Zn_0.98_Y_0.02_O, Zn_0.97_Y_0.03_O) as functions of temperature.

The temperature dependent electrical conductivity (*σ*) has been measured for all the series of pristine and Y-doped ZnO, as shown in Fig. 7(a). For all the series of pure and doped samples, there is a trend showing an increase in the electrical conductivity (*σ*) *vs. T*, depicting a semiconducting behavior. The maximum *σ* value of 6.50 S m^−1^ at 650 K has been recorded for Zn_0.98_Y_0.02_O (Y-2%) composition, which is about 100% higher than that of the pristine ZnO. It is evident from the *σ* plots that Zn_0.98_Y_0.02_O (Y-2%) is the optimum doping level, as for the doping content *Y* > 0.02, *σ* starts decreasing. The predominant reason for the decreasing in *σ* upon further increase in *Y* content may be associated with the increased defects in the lattice and further increase in the presence of secondary phase, *i.e.*, Y_2_O_3_. The presence of the secondary phase offers enormous carrier scattering to impede the movement of charges. As a result, (*σ*) starts decreasing upon further increasing the doping contents.^58,81^

The Seebeck coefficient of all the pure and Y-doped ZnO samples was measured under a temperature range of 300 K to 650 K. Fig. 7(b) shows that all the samples exhibit a negative value of the Seebeck coefficient, which confirms n-type semiconducting behavior of all the pristine and Y-doped samples. It can be seen that the Seebeck value for all the Y-doped ZnO samples has increased as compared to that of pure ZnO. The highest (*S*) value recorded is −506.30 μV K^−1^ at 625 K for Zn_0.99_Y_0.01_O (Y-1%), which is about 67% larger than that of the pure ZnO at the same temperature, which is the highest Seebeck value in rare earth dopants. Upon further increasing doping content, *Y* > 0.01, it can be seen that the (*S*) value decreases due to the synergetic effects of an increase in the carrier concentration (*n*) and the electronic specific heat (*C*_e_), as expressed by Mott's formula for degenerate semiconductors.^82^2
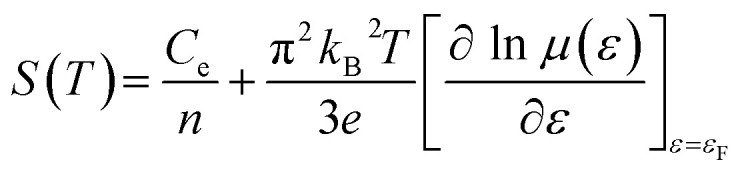
where, *μ*(*ε*) is the energy correlated carrier mobility, *e* is the electronic charge, (*k*_B_) is Boltzmann constant, and (*E*_f_) is the Fermi energy. Although (*S*) typically decreases with increasing *n* (as a result of Y-doping), (*C*_e_) is likely to increase, which is associated with the enhanced carrier scattering caused by the lattice defects as introduced by Y substitution into the ZnO lattice and the formation of Y_2_O_3_, which act as strong scattering centers.^42,83^

**Fig. 8:**
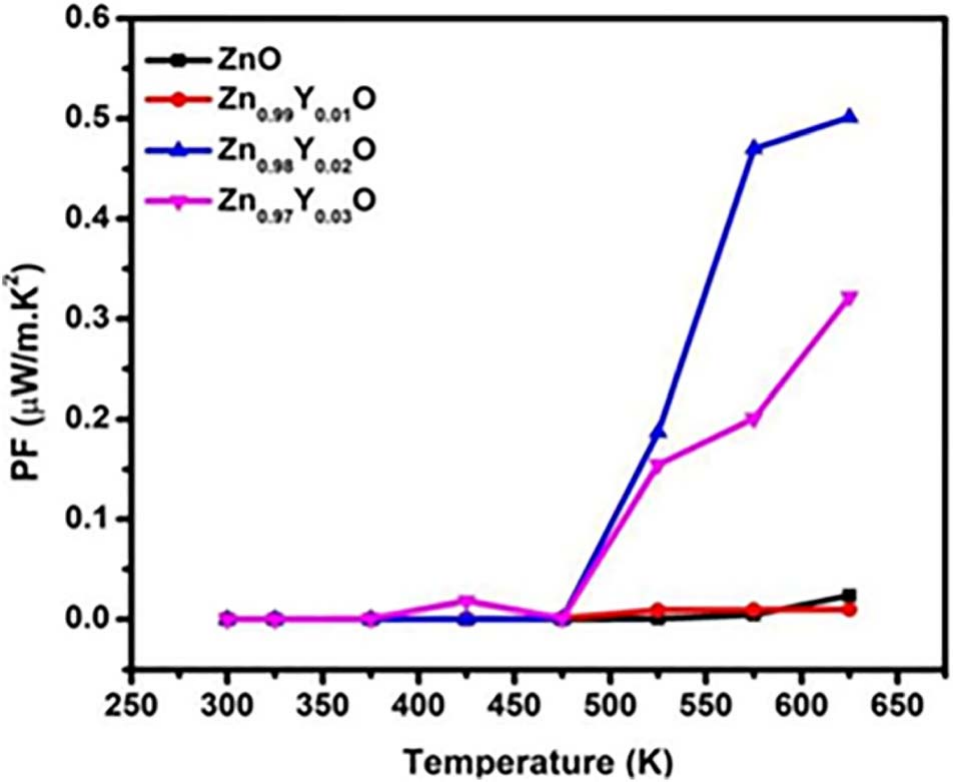
Temperature-dependent power factor (PF) of ZnO and yttrium-doped ZnO.

**Fig. 9:**
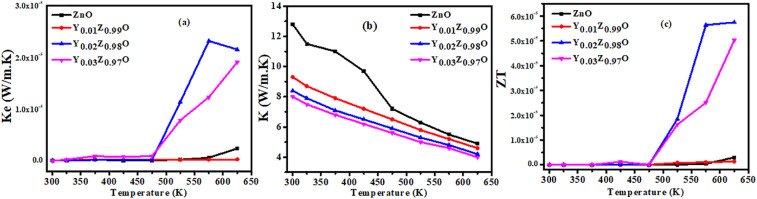
Electronic thermal conductivity (*K*_e_) and total thermal conductivity (*K*) (a) and (b), estimated (*ZT*) (c).

The temperature dependence power factor (PF = *S*^2^*σ*) for all the series of samples is shown in Fig. 8. The PF increases with increasing temperature for all compositions. The highest PF of 0.47 μW m^−1^ K^−2^ has been achieved for Zn_0.98_Y_0.02_O (Y-2%), which is about 100 times greater than that of the pure ZnO. The (PF) starts decreasing with further increases in Y-doping concentration, mainly due to enhanced carrier scattering from lattice defects and secondary phase formation. The obtained improvement in (PF) at an optimum doping level is mainly associated with the increased electrical conductivity. Therefore, Zn_0_._98_Y_0_._02_O is proposed as the optimum composition for this study. Fig. 9(a) shows the electronic contribution (*κ*_e_) to the total thermal conductivity, estimated *via* the Wiedemann–Franz relation *K*_e_ = *L*_o_*σT*, where *L*_o_ is the Lorentz constant, (*σ*) is electrical conductivity, and *T* is the absolute temperature.^84^ With increasing temperature, *κ*_e_ rises gradually following the trend of *σ*. The total thermal conductivity (*κ*_t_), as shown in Fig. 9(b), was estimated using *κ*_e_ from Fig. 9(a), along with the lattice thermal conductivity (*κ*_l_) adopted from the ref. 85*κ*_t_ of all the series of samples decreased upon increasing Y-doping content due to enhanced phonon scattering against the point-defects.

The dimensionless figure of merit (*ZT*) for all the series of composition has been estimated and shown in Fig. 9(c). Combined effect leads to calculated highest estimated value of *ZT* of Y-2%, which is 5.6 × 10^−5^ which is higher than pure and doped thin films as shown in Fig. 9(c). The highest *ZT* value has been achieved by the composition Zn_0.08_Y_0.02_O due improved PF and suppressed *κ*_t_.

## 4 Conclusion

In the present study, a facile sol–gel method followed by conventional sintering was employed to develop a series of pristine and Y-doped ZnO (Zn_1-*x*_Y_*x*_O; *x* = 0.01, 0.02, 0.03) ceramics for thermoelectric applications. XRD analysis revealed the formation of a secondary Y_2_O_3_ phase in all Y-doped samples, with its intensity increasing at higher doping concentration. The substitution of Zn^2+^ by Y^3+^ in the ZnO lattice contributes additional electrons, enhancing carrier concentration. This substitution also promotes grain growth and modifies the microstructure, which in turn influences the transport behavior. As the doping concentration increases, grain growth becomes more pronounced. Upon Y-doping, the electrical conductivity increases due to an increased carrier concentration up to an optimum doping level of Zn_0.98_Y_0.02_O, which is more prominent at higher values of temperature. The highest electrical conductivity obtained is 6.50 S m^−1^ at 650 K for Zn_0.98_Y_0.02_O. For all the series of pure and doped samples, the Seebeck coefficient values are negative, revealing n-type semiconducting behavior. After Y-doping, the Seebeck coefficient also increases for the series. As a result of increased electrical conductivity and Seebeck coeffcient, the highest power factor (PF) value of 0.47 μW m^−1^ K^−2^ has been achieved for Zn_0.98_Y_0.02_O, which is approximately 100-fold higher that of the pure ZnO. This increase mainly originates from the enhanced electrical conductivity. PF begins to drop upon further increase (*Y* > 0.02) in the doping contents. However, a further increase in *Y* content (*x* = 0.03) resulted in a decline in performance, likely due to excessive secondary phase formation and carrier scattering. Compared to previous reports on Y-doped ZnO *via* solid-state routes and Al/Ga-doped ZnO prepared by sol–gel, the present study shows superior power factor performance and more controlled secondary phase formation. The highest estimated *ZT* value of 5.6 × 10^−5^ has been achieved for the doped composition Zn_0.98_Y_0.02_O. Although long-term stability was not the primary focus of this study, the observed structural integrity and thermally stable phase up to 650 K indicate promising thermal durability. Moreover, the sol–gel method provides a low-cost, low-temperature synthesis route with potential for large-scale fabrication, making it attractive for industrial thermoelectric applications.

## Conflicts of interest

No conflict of interest declared by the author.

## Data Availability

Data will be provided upon request.
